# PHYSICAL ACTIVITY AND BODY FAT IN ADOLESCENTS LIVING WITH HIV: A COMPARATIVE STUDY

**DOI:** 10.1590/1984-0462/;2017;35;1;00012

**Published:** 2017

**Authors:** Priscila Custódio Martins, Luiz Rodrigo Augustemak de Lima, Davi Monteiro Teixeira, Aroldo Prohmann de Carvalho, Edio Luiz Petroski

**Affiliations:** a Universidade Federal de Santa Catarina (UFSC), Florianópolis, SC, Brasil.

**Keywords:** Physical activity, HIV, Adolescent

## Abstract

**Objective:**

To compare regular physical activity among adolescents living with human immunodeficiency virus (HIV) with their healthy peers, and to evaluate the relationship with anthropometric indicators of body fat.

**Methods:**

This was a cross-sectional study which investigated two groups: 57 adolescents (10-15 years of age) living with HIV, and 54 apparently healthy adolescents matched for sex and age. Physical activity was evaluated using a questionnaire and anthropometric measurements were performed. The groups were compared in terms of physical activity, and the linear and partial correlations (adjusted for age and sex) between physical activity and the anthropometric indicators were tested.

**Results:**

Adolescents living with HIV had a lower total activity score than their healthy peers (1.73 *versus* 2.14; p<0.001), but participated more frequently in physical education activities. Soccer and walking were the physical activities most frequently reported by adolescents of the two groups. No correlation was observed between total physical activity score and anthropometric indicators of body fat when adjusted for sex and age. Female gender (β=21.51), months of exposure to antiretroviral therapy (β=1.26), and socioeconomic classes B and C (β=22.05 and 28.15, respectively) explained 33% of the sum of skinfolds in adolescents living with HIV (F=6.70; p<0.001).

**Conclusions:**

Adolescents living with HIV have lower physical activity scores compared with their healthy peers, but physical education was found to be an opportunity to increase physical activity.

## RESUMO

**Objetivo:**

Comparar a atividade física habitual entre adolescentes que vivem com o vírus da imunodeficiência humana (HIV) e seus pares saudáveis e testar a relação com indicadores antropométricos de gordura corporal.

**Método:**

Estudo transversal, com dois grupos de investigação, composto por 57 adolescentes (10-15 anos) com HIV e 54 adolescentes aparentemente saudáveis, pareados por sexo e idade. Medidas antropométricas foram realizadas e a atividade física habitual foi obtida por um questionário aplicado em entrevista. Os grupos foram comparados de acordo com os exercícios físicos e foi testada a correlação linear e correlação parcial (ajustada por sexo e idade) entre atividade física e os indicadores antropométricos.

**Resultados:**

Adolescentes que vivem com HIV apresentaram menor escore total de atividade física, comparados aos pares saudáveis (1,73 *versus* 2,14; p<0,001); porém tiveram maior participação nas práticas de educação física escolar. Ambos os grupos praticaram futebol e caminhadas mais frequentemente, entre as atividades físicas relatadas. Não houve correlação entre o escore total de atividade física e os indicadores antropométricos de gordura corporal, quando ajustado por sexo e idade. As variáveis sexo feminino (β=21,51), meses de exposição à terapia antirretroviral (β=1,26) e as classes econômicas “B” e “C” (β= 22,05 e β=28,15, respectivamente) explicaram 33% do somatório de dobras cutâneas de adolescentes que vivem com HIV (F=6,70; p<0,001).

**Conclusões:**

Adolescentes com o HIV têm menor escore de atividade física comparados aos pares saudáveis, porém a educação física escolar se mostrou um espaço favorável para o aumento dessa prática.

## INTRODUCTION

Physical activity brings many health benefits and prevents, primarily and secondarily, degenerative complications of various diseases.^1^ At least 60 minutes of moderate to vigorous physical activity is recommended for children and adolescents. These activities may be accomplished in different formats and may occur in various contexts. This may be even more important for children and adolescents living with HIV, because in the long term exposure, the combined antiretroviral therapy (ART) may lead to dyslipidemia, insulin resistance, and accumulation of body fat in the trunk,^2^ which increases the risk of cardiovascular diseases.^3^

In adults with HIV, a meta-analysis found that aerobic, resistance or combined exercise, reduces body fat, increases lean body mass, as well as improves strength, aerobic fitness, symptoms of depression, and quality of life.^4^ In children and adolescents, only one study has shown that aerobic and strength exercises were safe and effective in increasing lean body mass, flexibility, aerobic fitness, and strength, however, without changing body fat and lipids.^5^ Reducing body fat, especially trunk fat, may lead to a reduction of the adverse effects of metabolic and cardiovascular changes^6^ besides promoting quality of life.

Children and adolescents with chronic conditions often have restrictions on participation in physical activity and sports owing to actual and perceived limitations.^7^ This leads to a reduction in physical fitness and functional capacity,^7^ which can be exacerbated by the evolution of HIV infection and by the ART.^8^ Low levels of regular physical activity were found in children and adolescents living with HIV;^9^ ^,^ ^10^ however, other studies concluded that most children and adolescents were engaged in regular physical activity.^11^ ^,^ ^12^ In matched case-control studies, no differences were found between patients living with HIV compared with healthy peers;^13^ ^,^ ^14^ however, these studies did not address physical activity as the main outcome. In addition, the instruments applied had significant limitations in capturing intensity, types, and contexts of physical activity. Data also represented only patients from developed countries.

The aims of this study were:

1. to compare the physical activity among adolescents living with HIV and their healthy peers;
2. to investigate the relationship between physical activity and anthropometric indicators of body fat in adolescents living with HIV, and making adjustments for possible confounding factors.

## METHOD

Observational and cross-sectional study including a control group for comparison, which investigated 57 adolescents of both sexes, aged 10-15 years, living with HIV acquired by vertical transmission, and in clinical follow-up at *Hospital Infantil Joana de Gusmão* (HIJG) in Florianopolis, Santa Catarina, Brazil. Inclusion criteria were:

- Presenting HIV infection by vertical transmission in medical records.
- Presenting clinical and laboratory evidence in the medical records (CD4^+^T cells, CD8^+^T cells, viral load, exposed or not to ART, type and duration of ART).
- Being able to remain standing and to communicate.

If the participant presented any pathology - except HIV infection - which altered body composition, he or she should be excluded from the study. Then, the control group was recruited pairing participants by sex and age in an allocation ratio of 1:1. Fifty-four healthy adolescents, who reported for the study had not been diagnosed with HIV and other diseases that alter body composition in the past six months, were part of the control group. After data collection, three participants had incomplete data on skinfold thickness as well as in the questionnaires, and this in turn hindered the analysis. All were students of a municipal elementary school in Florianopolis, Santa Catarina, located near the HIJG.

Regular physical activity was investigated by the Physical Activity Questionnaire for Older Children (PAQ-C),^15^ which is a questionnaire that enables the analysis of type, frequency, and intensity of activities from nine questions concerning the practice of sports, games, exercise in the school environment and during leisure time. The final score is determined by the arithmetic mean of the questions. A score equivalent to 1 to <3 was considered insufficiently active, and equivalent to ≥3 was considered active.^15^ The questionnaire was administered in an interview, and the investigated physical activities were related to the previous seven days. A validation study which used pedometers for seven days as the reference method showed that the PAQ-C is valid (r=0.35 to 0.43).^16^

The body weight, height, skinfold thickness, and body circumference measurements were obtained. For all the children, the procedures of the International Society for the Advancement of Kinanthropometry (ISAK) were followed. Body mass was measured with a digital scale Tanita^®^ (BF683W, Arlington Heigths, USA), with a capacity of up to 150 kg and resolution of 100 g. Height was measured using a stadiometer Sanny^®^ (ES2060, São Paulo, Brazil), with resolution of 0.1 cm. Body mass index (BMI) was calculated based on these measurements. Triceps, subscapular, abdominal, and calf skinfold thickness and perimeters of the relaxed arm and waist were measured with a Cescorf adipometer (Cescorf, Porto Alegre, Brazil) and anthropometric tape Sanny^®^, respectively. All measurements were obtained by raters who were calibrated and certified by ISAK.

The sociodemographic information on participants’ age, gender, and skin color, as well as family income and education of the parents or others responsible for the participant was collected in an interview. The economic level was determined according to the *Associação Brasileira de Empresas de Pesquisa*.^17^ Information on the stage of development of the disease, classified according to the Centers for Disease and Control (CDC),^18^ CD4 and CD8 T lymphocytes, HIV viral load, and antiretroviral therapy (treatment duration, drug type [classes], and adhesion) were obtained on the medical records of each patient.

Data were processed in Epidata program, version 3.1. Descriptive statistics were used for data analysis (mean, standard deviation, minimum and maximum, and frequencies). The Kolmogorv-Smirnov test and histograms were used to verify data normality. Student’s *t*-test for independent samples was used to compare physical activity and body fat between HIV^+^ and control groups. Pearson product-moment correlation and Spearman’s rank correlation were used to test the relationship between the score of physical activity and anthropometric indicators of body fat, for normal and asymmetric data, respectively. The partial correlation was tested with a control on the effect of sex and age. The magnitude of correlation between variables was interpreted according to the strength of the relationship (zero indicated no correlation; 0.01-0.39 indicated weak correlation; 0.40-0.69 indicated moderate correlation; 0.70-0.99 indicated strong correlation, and 1.00 corresponded to perfect correlation). For all analyses, we used the software STATA, version 11.0, establishing a value of *p*≤0.05.

This study was approved by the Ethics Committee in Research with Human Beings (CEPSH) of the *Universidade Federal de Santa Catarina* (CAAE: 34505314.7.0000.0121). The legal guardians signed an informed consent form to participate in the research. This study was in compliance with the Guidelines and Norms Regulating Research Involving Human Subjects of the National Health Council Resolution 466/12.

## RESULTS

This study included 111 adolescents, aged 10-15 years, distributed in HIV^+^ (n=57) and control (n=54) groups. In HIV^+^ group, most of the students were female (57.1%) and had white skin (29.8%), low income (56.1%), and low educational level (elementary education; 66.6%). In the control group, 51.9% of participants were female, predominantly white (57.4%), had low income (44.4%), and low educational level (59.3%). There were no significant differences in socio-demographic characteristics between the groups. Table 1 shows the anthropometric characteristics of the participants. Body weight, height, and arm circumference were lower in the case group compared with the control group, in contrast with the ratio of trunk to extremity skinfolds.

**Table 1:**
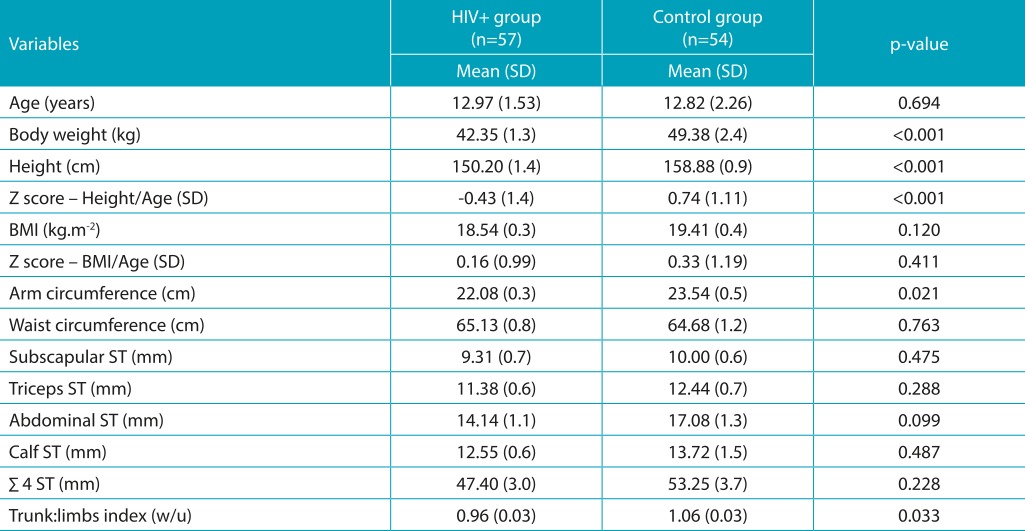
Anthropometric and sociodemographic characteristics of adolescents living with HIV and apparently healthy adolescents. Florianópolis-SC.

BMI: body mass index; ST: skinfold thickness; ∑ 4 ST: sum of subscapular, abdominal, calf, and triceps skinfold thickness. Trunk: limbs index: ratio of trunk to extremity; w/u: without unit. *Income: A (high class), B (middle class), C, D, and E (low class).

With regard to the clinical status of adolescents living with HIV, all adolescents aged below 13 years were in early stages of the disease, in asymptomatic or mild stage. For adolescents aged over 13 years, three had severe immunosuppression. Regarding the viral load, 31 (54.4%) adolescents had undetectable levels; 26 (45.6%) adolescents had detectable levels, six (10.5%) adolescents had viral load above 10 thousand copies, and two (3.5%) adolescents had viral load above 100 thousand copies. Other clinical and laboratory data are shown in Table 2.

**Table 2:**
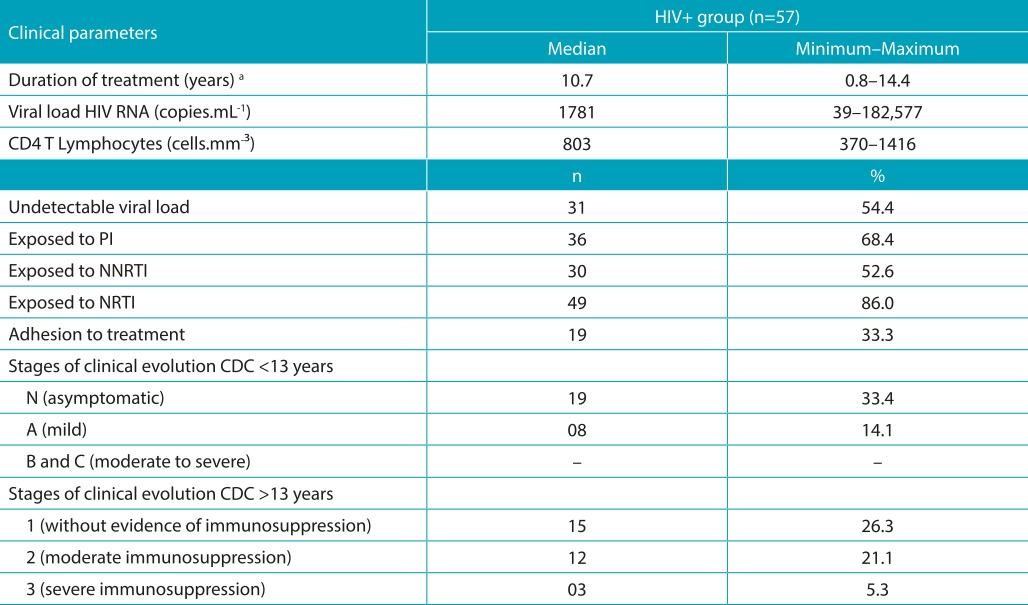
Clinical and treatment/infection parameters of adolescents living with HIV, Florianópolis-SC.

^a^Eight subjects did not undergo antiretroviral therapy. RNA: Ribonucleic acid; PI: Protease inhibitor; NNRTI: Non-nucleoside reverse transcriptase inhibitors; NRTI: Nucleoside reverse transcriptase inhibitors.

**Figure 1:**
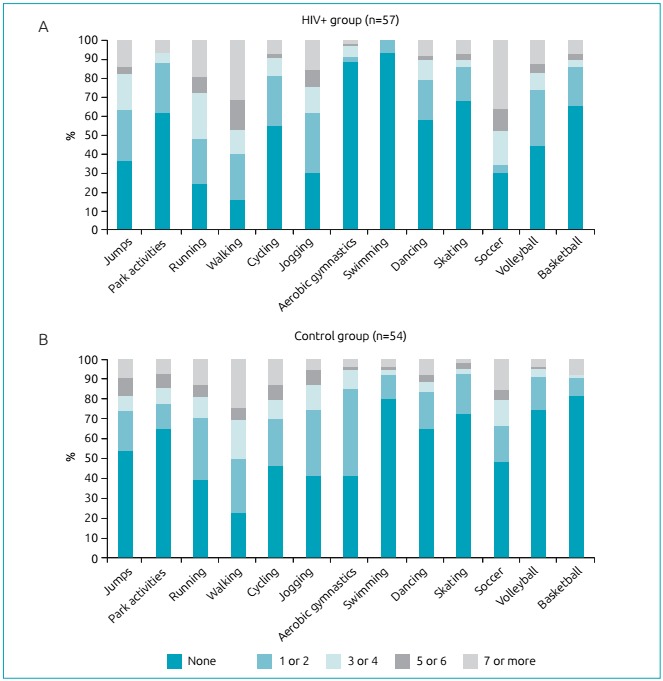
Weekly frequency and type of physical activities reported by adolescents living with HIV (A) and control group (B). Florianópolis-SC.

Fig. 1 shows the frequency and type of physical activity reported during the interview. Both groups performed similar activities; soccer and walking were the most frequent practices. In stratified analysis by sex, soccer and walking were the activities most performed by boys, whereas walking, running, and jogging were the most frequent activities among girls.

Table 3 presents the analysis of physical activity in both groups. Adolescents living with HIV had lower overall score of physical activity compared with the control group (*p*<0.001), although there is no difference in the activities performed during the week and on weekends. The prevalence of insufficiently active participants was 96.5% (n=55) for the case group, and 92.6% (n=50) for the control group. On an average, one-fourths of the sample does not perform any activity on weekends. On the other hand, more than half of the participants reported they always participate in physical education classes, and the greater proportion was among adolescents living with HIV, as compared to the control group (*p*<0.001). During school recess, both the groups were poorly engaged in physical activities, and 30% of them remained seated (talking, reading, or doing homework). Among the participants, 28% did not engage in physical activity on any day after the school.

**Table 3:**
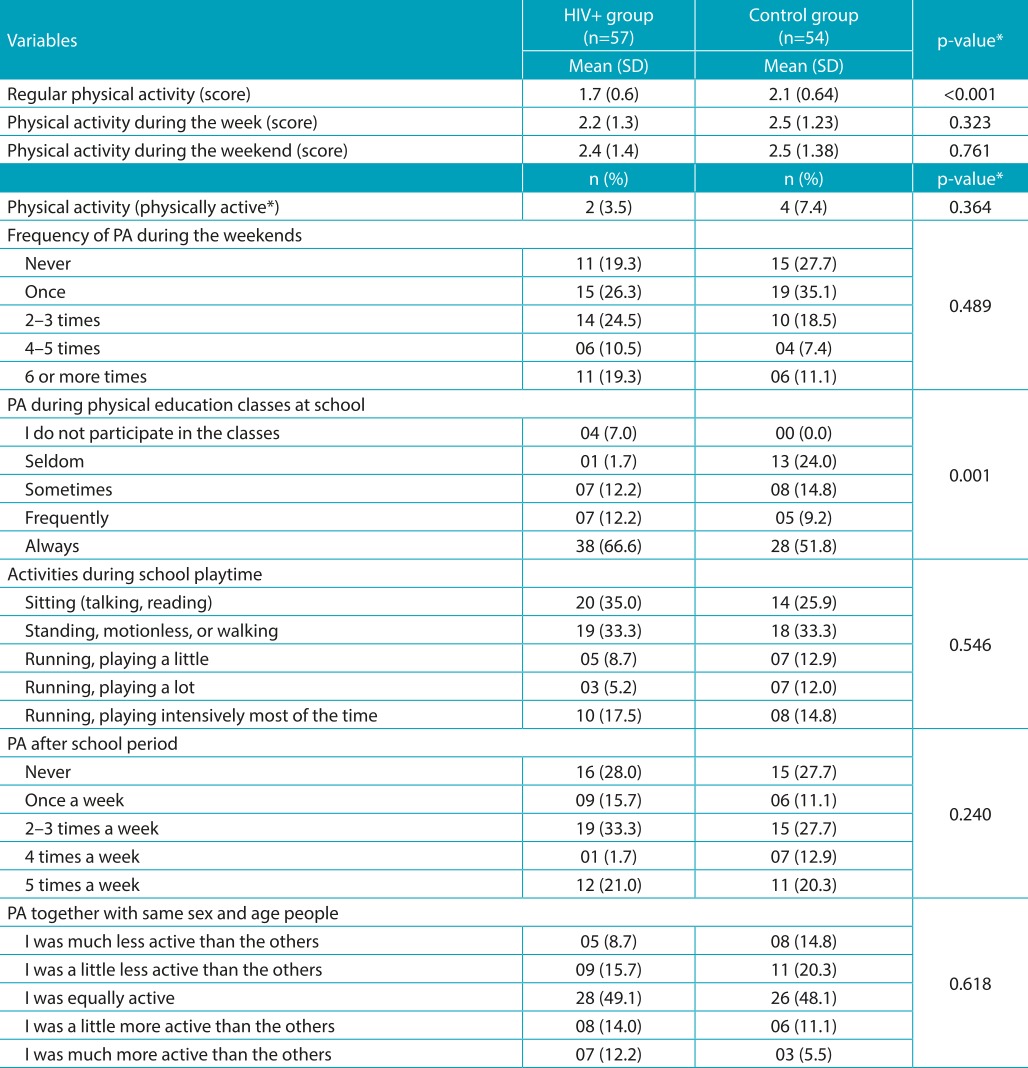
Analysis of the physical activity of adolescents living with HIV and their healthy peers. Florianópolis-SC.

PA: Physical activity; *Score ≤3 was considered as physically active.

In the bivariate analysis, there was an inverse relationship between the overall score of physical activity and body mass, abdominal skinfold, and the sum of skinfolds in adolescents living with HIV. However, when adjusted for the effect of sex and age, the correlations did not remain significant (Table 4). In addition, linear regression analysis was performed to test the conditions explaining the body fat variation, taking into consideration the sum of skinfolds. The female sex (β=21.51; *p*<0.001), months of exposure to ART (β=1.26; *p*=0.072), and economic classes “B” and “C” (β=22.05; *p*=0.103 and β=28.15, *p*=0.037, respectively) explained 33% of the variation in body fat in adolescents living with HIV (F=6.70; *p*<0.001). Years of exposure to ART showed an inverse relationship with the subscapular skinfold thickness (r=-0.29; *p*=0.04) and a positive relationship with body mass (r=0.42; *p*=0.01), height (r=0.36; *p*=0.04), and arm circumference (r=0.30; *p*=0.04). There were no differences in the triceps, subscapular, abdominal, and calf skinfold thickness as compared with HIV^+^ adolescents who used ART with protease inhibitors (PI) (n=36), ART without PI (n = 13), and did not use ART (n=8) (data not shown).

**Table 4:**
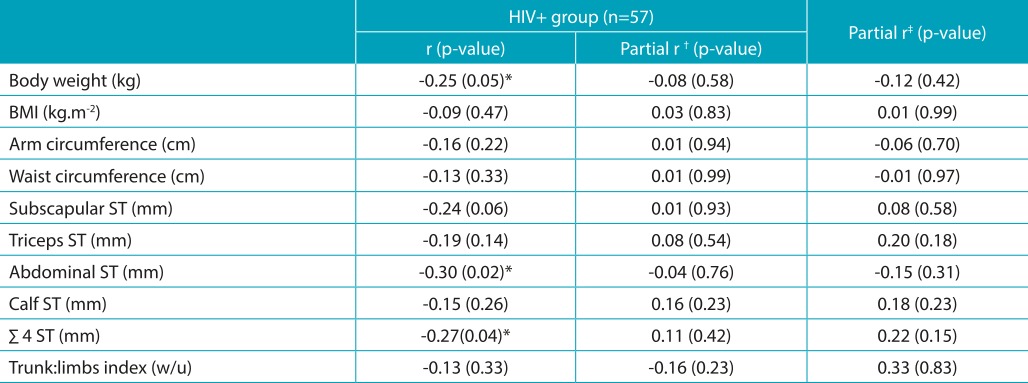
Correlation coefficients of the physical activity score and anthropometric indicators of body fat in HIV+ adolescents. Florianópolis-SC.

*p<0,05. Pearson product-moment correlation (body weight, height, BMI, and waist circumference) and Spearman’s correlation (arm perimeter and skinfolds) were used. ^†^Partial correlation adjusted for sex and age. ^‡^Partial correlation adjusted for sex, age, and ART duration. BMI: body mass index; ST: skinfold thickness; ∑ 4 ST: sum of the subscapular, abdominal, calf, and triceps skinfold thickness; w/u: without unit.

## DISCUSSION

The main finding of this study was to observe that adolescents living with HIV showed lower scores of physical activity when compared with those of the control group. However, they were more active in physical education classes. Participants of both groups were most often engaged in soccer and walking. The bivariate analysis showed that the relationship between physical activity and body fat in HIV^+^ group is poor (<0.40) and disappears when adjusted for sex, age, and exposure to ART. The sex, the time of exposure to ART, and economic level explained 33% of the sum of skinfolds in HIV^+^ group.

There is consensus in the literature that moderate to vigorous physical activity in healthy children and adolescents, performed for at least 60 minutes daily, brings benefits to cardiovascular health, decreases body fat, increases flexibility and muscle strength, in addition to bringing other psychosocial benefits.^19^ For children and adolescents living with HIV, there is no similar evidence. Although regular physical activity can be safe, beneficial, and effective in modifying the physical fitness in children and adolescents living with HIV, it was not enough to reduce body fat.^20^ In HIV^+^ adults, regular physical activity may be a tool to improve body composition.^21^

Lower scores of regular physical activity among adolescents living with HIV reinforce what was observed by the Specialized Care Service, which found a prevalence of 71.7% of adolescents who did not meet the recommendations on the practice of physical exercise.^9^ In a study carried out with South African HIV^+^ girls (aged 5-9 years), less time spent in vigorous physical activity and a lower participation in school physical education compared with healthy girls were observed.^22^ This reinforces the need to develop strategies to promote physical activity among this population and to understand the reasons why lower levels of activities have been found in the context of HIV. A literature review^23^ showed that social support from parents and friends are consistently and positively associated with physical activity levels of adolescents. Social isolation, stigma, and orphanhood related to HIV infection may be possible explanations.

In both groups, girls reported walking, running, and/or jogging, and boys played soccer. Similar to our study, there are reports that soccer and dance are the activities most commonly performed among boys and girls.^24^ In a study carried out in São Paulo, the activities most frequently performed by 91 HIV^+^ adolescents were soccer, volleyball, and cycling; however, the participants belonged to a broader age range.^25^ This reinforces that adolescents living with HIV perform the same activities as those apparently healthy, and therefore strategies to promote physical activity generally used can also be applied to improve the quality of life of HIV-infected people.

Although the lowest total score of physical activity has been found in this study, adolescents living with HIV showed increased participation in physical education classes. This is a mandatory curriculum subject regulated by Law No. 9,394, of 12/20/1996, which at least can partially contribute to children and adolescents to become more active, as they spend much of their day at school.^26^ Positive experiences with physical activity in childhood and adolescence can assist the individual to become a physically active adult.^27^ Moreover, the accumulation of short intervals of moderate to vigorous physical activity may be more feasible and effective to meet the recommendations on physical activity.^28^ Understanding why adolescents living with HIV are more active in school than outside school is difficult. Although the socioeconomic status is relatively similar in the investigated groups, the explanation may be associated with fewer opportunities for physical activities that require sophisticated physical space (club, gym, etc.) and professionals with remuneration. These variables were not collected and the hypothesis is only speculation; however, HIV infection is associated with pauperization in Brazil.^12^

Although several studies have shown an inverse relationship between regular physical activity and body fat in healthy adolescents,^29^ this was not observed in this study. The body composition of patients living with HIV is usually modified in the interaction between the virus, the ART, and the host. This change in phenotype was observed by the difference between the case and control groups, as well as by the contribution of ART (months of exposure) to the variation of the sum of skinfolds. Therefore, clinical and sociodemographic variables may represent in this context a stronger relationship with body fat than the physical activity. In HIV-infected adults, systematic physical activity has been considered a preventive intervention for the accumulation of visceral fat, because this accumulation at high levels is associated with chronic inflammation, metabolic disorders, and cardiovascular disease.^14^

Our hypothesis for the lack of correlation between physical activity and body fat indicators is based primarily in the process of growth and development of adipose tissue that HIV^+^ adolescents experience by the infection and exposure to ART. Adolescents living with HIV are generally shorter and have less body mass, as well as enter puberty late, when compared with their apparently healthy peers.^30^ This can be explained by mitochondrial toxicity caused by HIV and ART, psychosocial factors, deficiency in the intake and absorption of micronutrients, abnormal nitrogen balance, and impaired secretion of growth hormone owing to the inhibitory effect induced by viral proteins.^2^ Therefore, a different pattern and lower quantity of fat may be found among those adolescents, which can influence the relationship between fat and regular physical activity.

More than half of these adolescents have undetectable viral load, which reveal the adequate clinical status of patients and the efficacy of drugs, which consequently can provide better quality of life.^2^ On an average, adolescents had 10 years of exposure to ART, which is a long period; therefore, adverse effects such as insulin resistance, dyslipidemia, decreased bone mineral content, chronic inflammation, and atherosclerotic cardiovascular disease may occur.^3^ ^,^ ^14^ In addition, all adolescents in HIV^+^ group were infected via mother-to-child transmission, which also characterizes a long exposure to the virus. Therefore, strategies to minimize the adverse effects of long exposure to ART should be sought. These complications can decrease the quality of life of this population and interfere with regular physical activity,^28^ reinforcing the underactivity cycle.^7^ Owing to this study design, it is not possible to indicate cause and effect, and reverse causality may be considered.

Limitations of this study include the cross-sectional design, which encompasses the inability to establish cause and effect, the subjective measure of physical activity by questionnaire and indirect assessment of body fat. The strengths include selecting the sample from a referral hospital in the treatment of pediatric HIV and having a control group matched for sex and age.

We concluded that adolescents with HIV have lower scores of regular physical activity when compared with their healthy peers, although they have greater participation in physical education classes at school. The school environment can be a privileged and even more favorable space to promote physical activity among this population. Longitudinal studies are needed to confirm whether low levels of regular physical activity persist until adulthood. Sex, exposure to ART, and economic level explain a third of the sum of skinfolds in adolescents living with HIV. Although physical activity was not related to body fat indicators, studies with objective measures, which focus on metabolic health parameters that precede changes in body composition, are necessary.
